# Editorial: Advanced technologies for oral and craniomaxillofacial therapy

**DOI:** 10.3389/fbioe.2026.1759125

**Published:** 2026-01-12

**Authors:** Nicholas G. Fischer, Bolei Cai, Dan Lin

**Affiliations:** 1 Department of Bioengineering, University of Pennsylvania, Philadelphia, PA, United States; 2 Center for Precision Engineering for Health, University of Pennsylvania, Philadelphia, PA, United States; 3 Center for Innovation and Precision Dentistry, University of Pennsylvania, Philadelphia, PA, United States; 4 State Key Laboratory of Oral and Maxillofacial Reconstruction and Regeneration, National Clinical Research Center for Oral Diseases, Shaanxi Clinical Research Center for Oral Diseases, Department of Oral and Maxillofacial Surgery, School of Stomatology, The Fourth Military Medical University, Xi’an, China; 5 Intelligent Inspection and Diagnostics Health Service Platform, Shanghai University of Medicine and Health Sciences, Shanghai, China; 6 Shanghai University of Medicine and Health Sciences Affiliated Zhoupu Hospital, Shanghai, China

**Keywords:** artificial intelligence, biomaterials, dental materials, digital dentistry, interdisciplinary collaboration

Interdisciplinary collaboration is fundamental to advancing dental biomaterials research. Oral and craniomaxillofacial health challenges span the entire human lifespan and involve extraordinarily diverse tissues. From congenital craniofacial developmental anomalies to caries, periodontal disease, bone loss, and age-related degeneration, clinicians face continuous demands for intelligent, functional, and bioactive materials that regenerate, protect, or restore both soft and hard tissues across the craniofacial complex. Many of these clinical problems cannot be solved by any single discipline. For example, persistent gaps remain in producing dental materials with reliable anti-microbial, anti-fouling, abrasion-resistant, and self-healing properties, or engineering materials that can concurrently address infection, inflammation, aging, or osteoporosis. These needs highlight the necessity of integrating material science, oral biology, and clinical insights to design patient-specific solutions.

The mechanical and biological environment of the oral cavity is uniquely complex, with dynamic loading, fluid exposure, microbiome interaction, and rapid tissue turnover. Challenges such as uncontrolled force transmission during orthodontics or prosthodontics, unpredictable bone remodeling around implants, and biomaterial-related complications arise from the lack of precise biomechanical control and inadequate understanding of multiscale mechanisms. Interdisciplinary teams—uniting engineers, biologists, clinicians, and computational modelers—are essential for uncovering the cellular and molecular pathways involved in tissue integration. Such collaborations enable the development of immunoregulatory scaffolds, biomolecule-modified biomaterials, and next-generation polymers capable of resisting microbial colonization and mechanical wear.

As digitalization and artificial intelligence (AI) rapidly transform biomedical innovation, interdisciplinary collaboration again becomes crucial. AI-driven imaging, predictive modeling, and automated design pipelines now make it possible to optimize orthodontic biomechanics, personalize prosthodontic devices, guide surgical planning, and accelerate the discovery of new biomaterials. Similarly, digital dentistry workflows allow clinicians and engineers to refine therapeutic procedures, improving accuracy, efficiency, and patient outcomes. Together, these converging disciplines are redefining what is possible in oral and craniomaxillofacial therapy.

Here, in this Research Topic, we assembled a group of manuscripts that push the field forward through interdisciplinary research, including next-generation photothermal therapies to combat antibiotic resistance (Wang et al.), finite element analysis for optimizing orthodontic treatment (Zeng et al.), and advanced materials systems such as drug-loaded chitosan nanoparticles (Li et al.), imidazolate framework-8 composite nanofibrous membranes (Wang et al.), MXene nanomaterials (Li et al.), and self-healing resin composites (Han et al.). Furthermore, interdisciplinary efforts have yielded anti-cancer biomaterials (Wen et al. and Wen et al.), enhanced computer-assisted surgical systems (Wu et al., Bao et al., and Miadili et al.), refined surgical techniques for implant site preparation (López-Valverde et al.), and AI-aided decision support for orthodontic extractions (Huang et al.). These studies also address rising societal challenges, such as aging populations (Fu et al.), demonstrating the expansive impact of cross-disciplinary approaches (Zhou et al., Liu et al., and Wang et al.).

The studies collected in this Research Topic demonstrate how interdisciplinary collaboration is no longer optional but foundational to meaningful progress in dental and craniomaxillofacial biomaterials. By integrating engineering, biology, clinical expertise, and emerging digital and AI-driven technologies, and smart materials, these works demonstrate how complex challenges may be addressed more effectively than any single field could achieve alone. As the oral health landscape continues to evolve, such collaborations will remain essential for translating fundamental discoveries into innovative, patient-centered, personalized medicine solutions that elevate the standards of care across the lifespan.

